# Deleterious mutations show increasing negative effects with age in *Drosophila melanogaster*

**DOI:** 10.1186/s12915-020-00858-5

**Published:** 2020-09-30

**Authors:** Martin I. Brengdahl, Christopher M. Kimber, Phoebe Elias, Josephine Thompson, Urban Friberg

**Affiliations:** IFM Biology, Linköping University, Linköping, Sweden

**Keywords:** Aging, Deleterious mutations, *Drosophila melanogaster*

## Abstract

**Background:**

In order for aging to evolve in response to a declining strength of selection with age, a genetic architecture that allows for mutations with age-specific effects on organismal performance is required. Our understanding of how selective effects of individual mutations are distributed across ages is however poor. Established evolutionary theories assume that mutations causing aging have negative late-life effects, coupled to either positive or neutral effects early in life. New theory now suggests evolution of aging may also result from deleterious mutations with increasing negative effects with age, a possibility that has not yet been empirically explored.

**Results:**

To directly test how the effects of deleterious mutations are distributed across ages, we separately measure age-specific effects on fecundity for each of 20 mutations in *Drosophila melanogaster*. We find that deleterious mutations in general have a negative effect that increases with age and that the rate of increase depends on how deleterious a mutation is early in life.

**Conclusions:**

Our findings suggest that aging does not exclusively depend on genetic variants assumed by the established evolutionary theories of aging. Instead, aging can result from deleterious mutations with negative effects that amplify with age. If increasing negative effect with age is a general property of deleterious mutations, the proportion of mutations with the capacity to contribute towards aging may be considerably larger than previously believed.

## Background

Aging is the decline in physiological function with age, which results in a gradual decrease in survival and/or reproductive performance [1–4]. Aging affects many organisms and studies have shown that it has a genetic basis (reviewed in [5]). Theory suggests that aging evolves because the strength of selection declines with age [6–11], in combination with mutations with age-specific effects on performance. Two main evolutionary/genetic theories of aging have been proposed based on this idea. They both suggest that aging results from mutations with late acting deleterious effects, while they differ in that Mutation Accumulation (MA; [8]) assumes aging mutations to be neutral early in life whereas antagonistic pleiotropy (AP; [9]) assumes them to be beneficial.

The AP and MA theories of aging have both been tested extensively, and evidence in favor of each has been found in the laboratory as well as in the wild (AP, e.g., [12–18], MA, e.g., [19–29]). The two theories make contrasting predictions about how early and late life performances are associated with one another. While AP predicts a negative association, the initial formulation of the MA theory, based on mutations with effects spanning very short age intervals, predicts no association [2, 30]. These predictions stand in sharp contrast to the positive pleiotropy often observed between early and late life performance (e.g., [31–42]). Charlesworth [43] later extended the MA theory to also encompass mutations with effects spanning several (up to all) adult age classes, a modification which allows for positive pleiotropy under MA. Alternatively, aging can be reconciled with positive pleiotropy if variation in aging, at least within populations, is primarily caused by deleterious mutations with increasing negative effects with age. This possibility is also logically appealing, since from a biological perspective it is difficult to imagine a genetic architecture where mutations have deleterious effects exclusively confined to specific adult age classes. Aging through mutations with gradually increasing negative effects has rarely been considered (see discussions in [30, 33, 44, 45]), but is predicted by recent theory [46, 47].

The genetics of aging has primarily been studied using experimental evolution and quantitative genetics. These approaches have provided a general understanding of how genetic variants collectively contribute to aging, but provide limited information on the age-specific effects of individual mutations [48]. QTL studies and GWAS partly circumvent this limitation (e.g., [18, 28, 49–51]), but can generally only detect signals from mutations with large effects segregating at appreciable frequencies. To learn more about how the selective effect of individual deleterious mutations is distributed over age classes, we here measure the effect on female fecundity across adult life for 20 mutations in *Drosophila melanogaster*. From an evolutionary perspective, aging concerns both the elevation of mortality and the reduction in reproductive performance with age. Studying the latter however has several experimental advantages: as the same individuals can be measured at several time points, it provides a general measure of somatic condition, and experimental effort can be focused to key ages.

Our results show that 16 of the 20 tested mutations have a negative effect on fecundity and that the negative effect increases with age for 14 of these 16. Increasing negative effects with age could hence be an inherent property of deleterious mutations, causing most deleterious mutations to contribute towards aging.

## Results

To test if deleterious mutations have an increasing negative effect with advancing age, we independently introgressed 20 dominant mutations (*Bl[1]*, *Bsb[1]*, *bw[D]*, *Dfd[1]*, *Dr[1]*, *Frd[1]*, *Gl[1]*, *H[2]*, *Ki[1]*,* Kr[If-1]*, *L[rm]*, *Ly[1]*, *nw[B]*, *Pin[1]*, *Pri[1]*, *Pu[2]*, *Rap1[1]*, *Sb[1]*, *sna[Sco]*, *wg[Sp-1]*) by backcrossing for ≥ 11 generations into our outbred *Drosophila melanogaster* base population (Dahomey). To test for age-specific effects on reproductive performance, we then estimated the fecundity of 500–550 females of each mutation when expressed in the heterozygous state in young (day 5), middle-aged (day 19), and moderately old (day 33) individuals and compared this to the fecundity of 500–550 paired wildtype females (see the “Methods” section and Additional file 1: Fig. S1 for details).

**Fig. 1:**
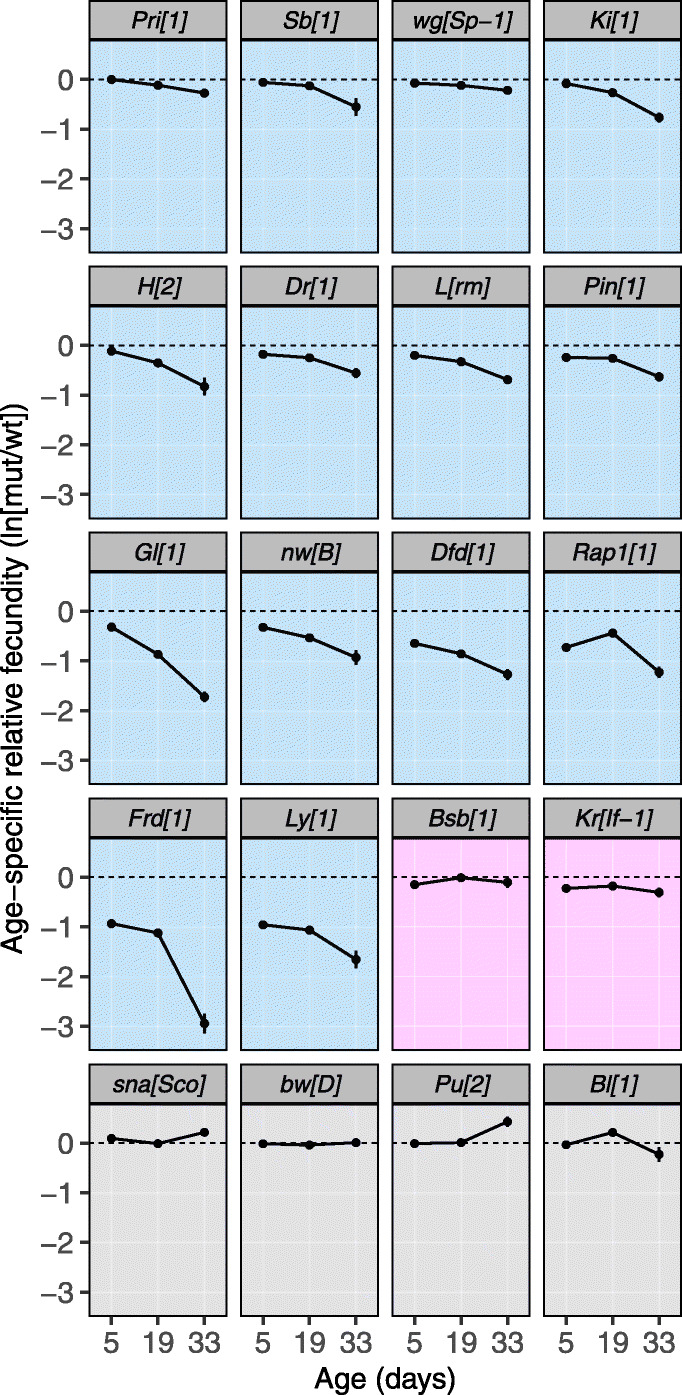
Relative fecundity of females carrying each of the different mutations at ages 5, 19, and 33 days old (mean ± SE). Blue background: mutations that are deleterious and have an increasing negative effect on fecundity with age. Pink background: mutations that are deleterious but do not show an increasing negative effect on fecundity with age. Gray background: mutations for which no deleterious effect was detected. Mutations are ordered after their effect on fecundity at day 5, separately for each category/color

Since our focus was on the effect of deleterious mutations on aging, we first asked which of the 20 mutations are deleterious with respect to fecundity. Analyzing fecundity data from young flies (5 days old) only, we find that 15 of the 20 mutations have a significantly deleterious effect, while one additional mutation had a significant deleterious effect when we also took fecundity at days 19 and 33 into account (Fig. 1, Additional file 2: Table S1).

By fitting a generalized linear mixed model (GLMM) with age as a covariate across the three age-specific measures of fecundity (as mutant versus wildtype egg counts at the vial level), we next tested if the deleterious mutations had an increasing negative effect on fecundity with age and find that this is the case for 14 of the 16 deleterious mutations (Fig. 1, Additional file 2: Table S2). As such, these 14 mutations increase the rate of reproductive aging. We also tested if we could detect an increasing negative effect when only comparing the effect on fecundity between two ages at a time (days 5 to 33, 5 to 19, and 19 to 33) using a similar model with age as a factor and find largely similar results (Additional file 2: Table S3).

**Fig. 2:**
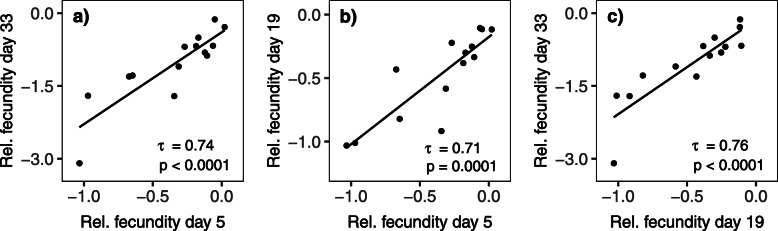
Correlations between relative fecundity (ln [mut/wt]) estimated at different ages. **a** Day 5 vs. day 33, **b** day 5 vs. day 19, and **c** day 19 vs. day 33, for the 14 deleterious mutations with increasing negative effects with age. Associations were tested with Kendall’s tau. Two-tailed *p* values are reported

Since several studies have suggested that both standing and mutational genetic variation show positive pleiotropy in their effects on life history traits between ages (e.g., [31–42]), we next tested if this was true for the 14 deleterious mutations that elevate the rate of aging studied here (using Kendall’s Tau which tests for an ordinal association between variables). Our results show a strong positive correlation in relative fecundity (ln[mut/wt]) between days 5 and 33, 5 and 19, as well as between 19 and 33 (all *p* ≤ 0.0001; Fig. 2). Similarly, strong positive correlations are also found when all 16 deleterious mutations are included (5 to 33: *τ* = 0.62, *p* = 0.0005; 5 to 19: *τ* = 0.62, *p* = 0.0005; 19 to 33: *τ* = 0.80, *p* < 0.0001). We also tested if relative fecundity at day 5 correlated to relative survival at day 33 and again find evidence for positive pleiotropy (*τ* > 0.51 and *p* < 0.005, irrespectively of whether we use the 14 aging inducing or the 16 deleterious mutations; Additional file 2: Fig. S2).

We were next interested in testing if the rate of aging induced by a mutation is associated with its degree of deleteriousness. To do this, we calculated the change in relative fecundity for each mutation between two ages at a time (old–young), and then tested if this difference was associated with the relative fecundity of the mutant at the first of the two ages using Kendall’s Tau. To be conservative, we first performed this test using all 16 deleterious mutations, regardless of whether they induced significant aging. We find no support for such an association when studying early-life aging (days 5 to 19: *τ* = − 0.10, *p* = 0.63), but when studying aging from early to late life (days 5 to 33: *τ* = 0.40, *p* = 0.03) and late-life aging (days 19 to 33: *τ* = 0.52, *p* = 0.005), we find that the deleteriousness of a mutation is associated with the rate of aging it induces. We find qualitatively similar results when analyzing only the 14 mutations that induced significant aging (results presented in above order: *τ* = − 0.12, *p* = 0.59; *τ* =0.47, *p* = 0.02; *τ* = 0.43, *p* = 0.04).

**Fig. 3:**
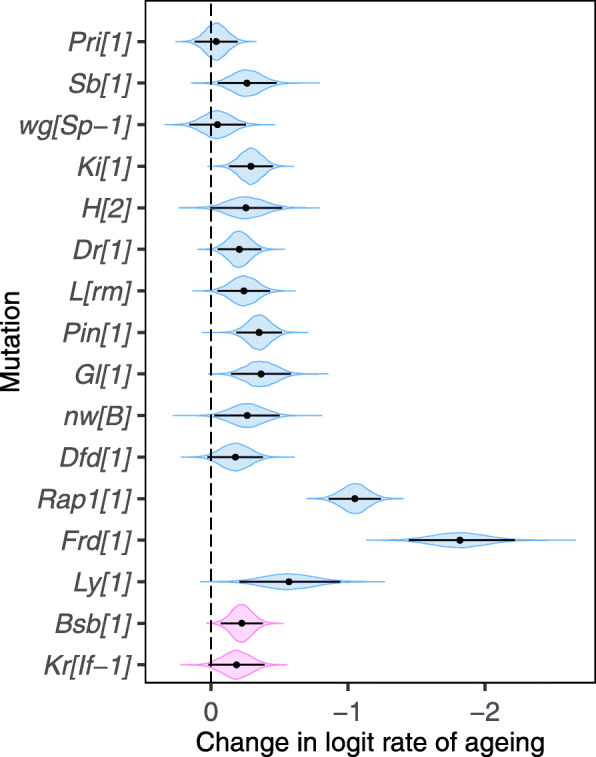
Difference between the rates of aging between the intervals 5 to 19 and 19 to 33 days of age (posterior mean, 95% credibility interval and posterior density distribution displayed). The dashed line indicates a constant rate of aging and values to the right indicate acceleration in the rate of aging with age. Deleterious mutations that increase the rate of aging are displayed in blue, while deleterious mutations that do not increase the rate of aging are displayed in pink

If the rate of aging induced by a mutation at a particular age is positively associated with its deleteriousness at that age, this in turn suggests that the rate of aging a mutation induces should accelerate with age in a self-reinforcing process. We find strong support for this hypothesis as 10 of the 14 deleterious aging inducing mutations show significantly higher rates of aging between days 19 and 33 compared to between days 5 and 19 (Fig. 3). In addition, the other four mutations, as well as the two that are deleterious but do not induce aging when taking all three ages into account, all have (non-significant) point estimates (posterior means) pointing towards faster aging later in life (Fig. 3).

**Fig. 4:**
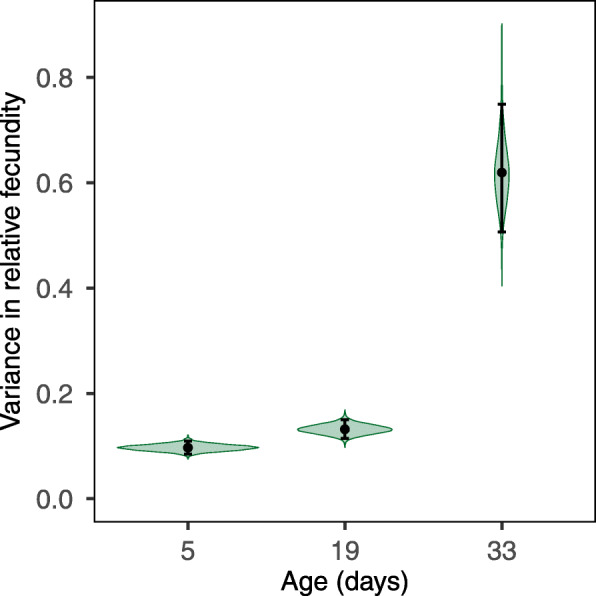
Variance in relative fecundity across the 16 deleterious mutations at 5, 19, and 33 days of age (posterior mean, 95% credibility interval and posterior density distribution displayed)

Evolution of aging through changes in the relative frequency of mutations that show increasing negative effects with age and positive pleiotropy across ages does not necessarily follow from changes in how the strength of selection declines with age. For this to follow, the age specific variance in fitness induced by these mutations must increase with age [2, 30, 33]. Our finding that deleterious mutations cause the rate of aging to accelerate with age suggests that this is the case, which a direct test for changes in the genetic variance in relative fecundity among mutations with age also confirmed (Fig. 4). The genetic variance increased both between day 5 and 19 (*p*_mcmc_ < 0.007) and between days 19 and 33 (*p*_mcmc_ < 0.001), irrespective of whether all 16 deleterious mutations or only the 14 aging inducing ones are included.

## Discussion

It is the declining strength of selection with age that creates the potential for evolution of aging, but for aging to actually evolve, mutations with age-specific effects are also required. The discussion of genetic variants with such properties has hitherto almost exclusively been limited to those assumed by the AP and MA theories of aging, while deleterious mutations with increasing negative effects with age have rarely been considered. Their shape in terms of age-specific effects is nevertheless conceptually very similar to those assumed by both the AP and MA theories: all three classes of mutations have effects that become worse with age, but differ in that they start out as either beneficial, neutral or deleterious in early life. In this study, we set out to test for age-specific effects of 20 presumably deleterious mutations. For 16 of these, we could detect a negative effect on fecundity and for 14 of these the negative effect increased with age. This result suggests that increasing negative effects with age could be a general property of deleterious mutations and that the pool of mutations contributing towards aging may be larger than previously thought.

If deleterious mutations in general induce genetic variation in aging, this property is not enough for these mutations to respond to a steeper decline in the strength of selection with age and cause evolution of faster aging. For this to follow, it is required that their increasing negative effect with age depends on how deleterious they are and thus that the variance in age-specific fitness they cause increases with age [30]. Our results suggest that deleterious mutations, in general, have this property, which implies that aging could evolve through these mutations in relation to age-specific intensities of extrinsic mortality, similarly to what is predicted when aging evolves through MA and AP mutations [30].

While this study provides direct support for the existence of deleterious mutations with increasing negative effects, many previous studies have given indirect support for allelic variants with these properties. Developments of the MA theory of aging [19, 52] predict that the genetic variance in fitness and inbreeding depression should increase with age and that “hybrid” vigor of individuals from crosses between different populations with small effective population sizes should be primarily confined to late ages. These predictions have been supported by a suite of studies (e.g., [19–25, 52–54]). However, provided that deleterious mutations with increasing negative effects show increasing variance in effect size with age, and that they in general are at least partly recessive, the above findings are also compatible with aging through deleterious mutations with increasing negative effects. Additional findings that are compatible with aging through this class of mutations are extended lifespan in populations that have experienced intensified selection on early adult performance [35, 40, 42], strong correlations between mutations reducing early adult fitness and lifespan [33, 41], and identification of mutations with increasing deleterious effects in a GWAS [50].

The few studies that have directly aimed at measuring age-specific effects of spontaneous mutations in *D*. *melanogaster* have, at least at face value, found results that differ from those reported here. Pletcher et al. [55, 56] studied a set of mutation accumulation lines and found that the variance in mortality rate among lines was larger early compared to late in life. Similarly, Yampolsky et al. [57]) and Mack et al. [58], assaying a different set of mutation accumulation lines, found that the effect of spontaneous mutations is larger early compared to late in life for mortality and fecundity, respectively, at least at early generations of mutation accumulation. These findings are also corroborated by theory, extending Fisher’s geometric model for adaptation to different ages [59]. There are however several viable alternative explanations to these results, including ones that suggest that the true effect of spontaneous mutations actually increases with age. Of the alternatives, heterogeneity in frailty [60, 61] among individuals within lines is the strongest candidate. The mutation accumulation lines studied by Pletcher et al. [55, 56] were all derived from an inbred line. Inbred individuals are known to be particularly sensitive to environmental perturbations [52, 62], which may have been further exacerbated since flies carried the mutation ebony. Variation between genetic lines could hence have been artificially reduced at late ages due to selective deaths of frailer individuals [63, 64]. The diminishing effect of reproduction on mortality at late ages could also have contributed to the estimated reduction in variance at old ages [65]. The studies by Yamplosky et al. [57] and Mack et al. [58] accumulated mutations through the Middle Class Neighborhood design, which causes individuals within lines to vary in their mutational load in addition to environmentally induced heterogeneity. A smaller departure of mutation accumulation lines from control lines (in mortality rate and fecundity) at late compared to young ages could hence have followed from selective death of individuals more heavily laden with mutations [57]. Our study largely avoided the potential problem of heterogeneity, since we studied fecundity only up until an age most flies survive to.

To be able to detect a signal of individual mutations in our experiments, we were constrained to focus on mutations with relatively large effect sizes. From this perspective, the mutations we assayed are not representative of spontaneous mutations, which in theory also could explain why our results differ from those of spontaneous mutations mentioned above. We cannot however see any reason why the age specificity of a mutation should depend on its effect size. This said, mutations with effect sizes comparable to those studied here would probably not contribute much to aging at the population level, since they should be rapidly purged from natural populations.

The negative effects of a deleterious mutation could plausibly increase with age because the effect of the mutation itself induces an increase in the degradation of somatic quality (either early in or continuously across life), altering the context in which the mutation’s effects in later life are felt. This could be realized through several different potential mechanisms. One possibility is that deleterious mutations commonly interfere with the efficiency of biochemical pathways and elevate the production of harmful/toxic byproducts. If some of these byproducts gradually accumulate with age, they could have an increasing detrimental impact with age and cause aging [44, 45, 66, 67]. Another possibility is that deleterious mutations reduce the robustness (i.e., quality/redundancy) of individuals. With imperfect repair systems, wear and tear would then result in faster failure of individuals carrying more deleterious mutations (for similar arguments see [68, 69]). In line with this scenario, environmental stress during early development hastens reproductive aging [70]. Yet, another possibility is that the adverse effect of deleterious mutations on organismal function and capacity to acquire resources results in fewer resources available for somatic repair. Deleterious mutations would then cause aging similarly to how aging is generated according to the disposable soma theory (a physiological account of AP), which states that aging follows from insufficient resources being invested into somatic maintenance due to strong selection for resource allocation into current reproduction [71, 72]. An extension of this argument is that individuals experiencing the negative effect from a deleterious mutation will age faster because they allocate relatively more resources into early reproduction in response to an elevated risk of death. Alternatively, it is possible that the negative effects of deleterious mutations do not increase with age per se, but that their impact is amplified with age because traditional aging mutations (MA or AP) cause the soma to be more sensitive to their effect at older ages [45]. This argument is similar to the one that the selective effect of deleterious mutations is larger in stressful environments, which has gained some empirical support [73] but does not seem to be generally true [74, 75]. In any case, even if the increasing negative effect with age of deleterious mutations depends on other mutations initiating aging, deleterious mutations will contribute to aging in the presence of any AP or MA mutations.

## Conclusions

The evolution of aging ultimately requires genetic variants with deleterious late-life acting effects. If these mutations primarily have beneficial or neutral effects early in life has been vividly debated, while mutations already expressing smaller deleterious effects early in life have only rarely been considered [30, 33, 44–46]. Our study does however demonstrate that deleterious mutations indeed can have negative effects that amplify with age and hence that increasing negative effect size could be a common property of deleterious mutations. Expressing a deleterious effect early in life exposes these mutations to negative selection to a much higher extent than those having a neutral effect early in life, keeping them at a considerably lower frequency, on a per locus basis, at mutation-selection-drift balance. The genome-wide influx of this type of aging mutation may however largely exceed those suggested by established aging theories, since logic suggests that most deleterious mutations should already manifest their harmful effect early in life, as most genes are expressed throughout life and show little expression dynamics during adulthood [76, 77]. If future studies on a wider diversity of mutations corroborate our findings, the pool of mutations we know to be contributing to senescence will be substantially expanded.

## Methods

### Fly population and mutations

We used flies from a laboratory-adapted population of *Drosophila melanogaster* known as Dahomey in our experiments, collected from the wild in 1970. Since then, it has been maintained as a large outbred population, housed in population cages with overlapping generations and under constant conditions (12 L∶12D light cycle, 25 °C, 60% relative humidity, and a standard yeast/sugar-based food medium), which we maintained during our experiments. Due to its population size (not strictly controlled but in the thousands) and overlapping generations, the Dahomey population has become widely used in studies of aging and lifespan (e.g., [15, 78–86]).

Twenty autosomal mutations (*Bl[1]*, *Bsb[1]*, *bw[D]*, *Dfd[1]*, *Dr[1]*, *Frd[1]*, *Gl[1]*, *H[2]*, *Ki[1]*, *Kr[If-1]*, *L[rm]*, *Ly[1]*, *nw[B]*, *Pin[1]*, *Pri[1]*, *Pu[2]*, *Rap1[1]*, *Sb[1]*, *sna[Sco]* and *wg[Sp-1]*) were included in this study. They were chosen on the basis that they had a reliable dominant visible phenotype, to make it possible to efficiently backcross them into our base population, and based on that, several of them have successfully been used earlier to address the effect of deleterious mutations on various evolutionary processes [87–90]. The mutations were obtained from Bloomington Stock Center and introgressed separately into the Dahomey genetic background for at least eleven generations of backcrossing. During the first two generations, mutant males were crossed with virgin Dahomey females; thereafter, virgin mutant females were crossed with Dahomey males. From generation F4 and onwards, the backcrosses consistently involved at least 100 virgin females paired with 50 males per mutation line (Additional file 1: Fig. S1A).

Prior to use in our experiments, the mutation lines were reared at controlled densities (~ 180 eggs per vial) for two generations; eggs in each vial were laid by 15 Dahomey virgins mated with 15 males heterozygous for the mutant. To sample a range of Dahomey haplotypes, this was replicated across 10 vials per line for the first generation, and then increased to 30 vials for the generation producing experimental flies. With this design, no maternal effects should have influenced our results, since mutant and wildtype flies all shared the same mothers. Any paternal effects [91] should have been equally shared between mutant and wildtype flies as they, for each line, had the same fathers (Additional file 1: Fig. S1B).

### Experimental design

We measured age-specific fecundity in four experimental blocks, each including five different mutations. We housed flies in vials containing 33 mutant and 33 wildtype females along with 33 males marked with *ebony* (introgressed into the Dahomey background), using 20 to 22 vials for each mutation. Flies were transferred to new vials four times a week throughout the experiment. At three equally spaced ages (day 5, 19, and 33 of adult life), we randomly separated out 25 females of each type from every vial to lay eggs for 24 h; mutant and wildtype females were separately housed in small bottles with lids made of petri dishes filled with standard medium as egg-laying substrate. Petri dishes were replaced after ~ 18 h to simplify counting. In cases when fewer than 25 females of either type were alive in a vial, all available flies of the type with fewest survivors were selected to lay along with a matching number of randomly selected individuals of the other type. During the laying period, all excess females and the males from each vial were retained, and at the end of the laying period, all flies originating from an experimental vial were placed back together. Eggs laid on both petri dishes from each vial were counted and subsequently used to estimate the relative fecundity for each mutation at each time point. *Ebony* males were replaced with younger males (~ 4 days of adult age) at days 13 and 27 to standardize the influence of males across the different ages female fecundity was assayed at (Additional file 1: Fig. S1C).

### Statistical analysis

All statistical analysis was performed in the R statistical environment [92]. All generalized linear mixed-effect models (GLMMs) were fit using Bayesian Hamiltonian Markov chain Monte Carlo via the rstanarm package [93]. We used weakly informative, normally distributed priors for both the intercept (mean = 0, SD = 10) and the coefficients (mean = 0, SD = 2.5), with autoscaling for the coefficient priors. Each model was run with four chains of 4000 iterations each and the first 1000 discarded as warm-up, and chain convergence was evaluated using the Gelman-Rubin potential scale reduction factor. Generally speaking, we then used the posterior distributions of model coefficients to calculate relevant posterior distributions that allowed us to test hypotheses of interest. We report the equivalent to a two-tailed *P* value (p_mcmc_) for these tests, calculated as two times the proportion of samples in a posterior distribution that was smaller/greater than the critical value (0), whichever was smaller.

We tested whether mutations were deleterious using a GLMM with a Poisson error distribution and log link function, with the syntax: fecundity ~ type * mutation + (1|ID). This model was run twice, once where fecundity was calculated only for day 5, and once where fecundity was summed across days 5, 19, and 33. Type and mutation are fixed factors representing mutant or wildtype females for each mutation line respectively, their interaction is a fixed factor, and ID is a random factor representing the vial they were housed in.

To test whether mutations cause aging, we used a model that directly compares the number of mutant eggs to wildtype eggs from females in each vial as a binomial response. This model took the form of a GLMM with a binomial error distribution and logit link function, with the syntax: (mutant fecundity|wildtype fecundity) ~ age * mutation + (1|ID), where age in days is modeled as a covariate, mutation and its interaction with age are fixed factors representing each mutation line, and ID is a random factor representing vial.

We also fit a very similar model to the one described above where age was modeled as a fixed linear factor. This model enabled us to test directly for differences in aging during each discrete interval between fecundity measures. The posterior distributions of coefficients from this model were also used for two additional tests. We first tested whether the rate of aging accelerated with age by calculating a posterior distribution for the difference between the rate of aging in the day 19–33 interval and the rate in the day 5–19 interval and then testing whether or not this difference was different from 0. We then tested whether variance among mutations increased with age by calculating the posterior distribution of among-line variances at each of days 5, 19, and 33 and then testing whether the difference in variance between each pair of ages was different from 0.

When testing whether there is positive pleiotropy in deleteriousness between ages, we first calculated the median relative fecundity (ln[mut/wt]) for each mutation across all replicate vials at each age (adding 1 to all egg counts to account for the occasional cases when no egg was laid). We then tested for a correlation (in the form of an ordinal association) between medians for each pair of ages using Kendall’s Tau. To test if there is a relationship between a mutation’s deleteriousness and the increase in rate of aging it induces, we first calculated the difference in median relative fecundity between each pair of ages for each mutation (ln[(mut_t+1_/wt_t+1_)/(mut_t_/wt_t_)] = ln[mut_t+1_/wt_t+1_] − ln[mut_t_/wt_t_]). We then tested for a positive correlation between this difference and the median relative fecundity at the first age using Kendall’s Tau. We note that this test is conservative, since random error in the estimate of the relative fecundity of the mutant at time *t* will contribute with the opposite effect on the difference in relative fecundity of the mutant between time points *t* and *t*+1, biasing the association towards negative values.

## Supplementary information

**Additional file 1: Figure S1.** Summary of experimental procedures. (A) Crosses used to introgress a dominant mutation into Dahomey, our outbred long-term laboratory adapted population. Top row shows male genotypes and bottom row female genotypes used in each cross. Males and females are depicted with their three main chromosomes (sex chromosomes at the top and major autosomes below) with the 4th dot chromosome omitted for brevity. We first crossed a male carrying the mutation (depicted with a horizontal line and a star) balanced over a balancer chromosome (black) to females from Dahomey (its genome colored in blue). From this cross we took mutant sons that we mated to Dahomey females. From the next cross onwards we took mutant daughters which we crossed to Dahomey males. This procedure was repeated in parallel for all 20 mutations, and replaced the mutations’ original genetic background (orange) with that of Dahomey. (B) Cross to produce focal mutant and wildtype females used in the experiment (observe that mutant and wildtype females were produce by the same parents). (C) Relative age-specific fecundity of each mutation was tested by hosting 33 mutant and 33 wildtype females with 33 males (marked with ebony [*e*]) in a single vial. Flies were transferred to a fresh vial every 1–2 days. At day 5 of adulthood 25 mutant and 25 wildtype females were randomly sorted out under light CO_2_ anesthesia and placed in separate flasks with a lid filled with food on the inside. Excess females and males were stored in separate vials. The 25 mutant and wildtype females laid eggs in the bottles over a 24 h period (food was replaced once). After egg-laying all flies were placed into a common vial and again transferred to fresh vials every 1–2 days. This procedure was repeated over five weeks, with fecundity measures taken 3 times 2 weeks apart. Males were replaced at a regular interval with respect to the fecundity assays, so that females had experienced males of the same age before each fecundity assay.

**Additional file 2: Figure S2.** Correlation between relative fecundity at day 5 (ln [mut/wt]) and relative survival at day 33 (ln[mut/wt]). Circles indicate deleterious mutations with an increasing negative effect on fecundity with age, triangles indicate deleterious mutations without an increasing negative effect on fecundity with age, and squares indicate mutations for which no deleterious effect on fecundity could be detected. Associations are tested with Kendall’s tau (τ > 0.51 and *p* < 0.005, irrespective of whether we use the 14 aging inducing, the 16 deleterious, or all 20 mutations). **Table S1.** Difference in relative fecundity between wildtype and mutant (*s* = 1 - mut/wt) for each mutation, either only taking early-life fecundity into account, or summing over all three ages fecundity measures were taken from. Estimates are presented together with their 95% credibility interval and associated *p*-value. The estimation was done in a GLMM with a Poisson error distribution and log link function; positive values indicate a deleterious effect of the mutation. **Table S2.** Estimated coefficient for the rate of aging on fecundity for each mutation (*Coeff*), presented together with 95% credibility interval and associated p-value. The estimation was done in a GLMM with a binomial error distribution and logit link function where age was treated as a covariate. Negative values indicate faster aging for the mutation compared to wildtype. **Table S3.** Aging, estimated as the relative difference (*Diff* – negative estimates indicate faster mutant aging) in fecundity between mutant and wildtype between two time points, along with 95% credibility interval and associated p-value. The estimation was done in a GLMM with a binomial error distribution and logit link function where age was treated as a factor. **Table S4.** Summary of the number of females [minimum, median, maximum] assayed for fecundity per vial for each time point. Note that we always assayed the same number of mutant and wildtype females from a vial at each time point. *For each of *Kr[If-1]* and *H[2]*, one vial was mistakenly started with fewer than 33 mutant and wildtype females.

## Data Availability

The dataset generated and analyzed during the current study are available at the Dryad repository [94].
