# Supplementary material for: Deleterious mutations show increasing negative effects with age in Drosophila melanogaster
Source: BMC Biol. 2020 Sep 30;18:128. doi: 10.1186/s12915-020-00858-5 (PMC7526172; doi:10.1186/s12915-020-00858-5)
Supplement: Supplementary file 1 — Additional file 1: Figure S1. Summary of experimental procedures. (A) Crosses used to introgress a dominant mutation into Dahomey, our outbred long-term laboratory adapted population. Top row shows male genotypes and bottom row female genotypes used in each cross. Males and females are depicted with their three main chromosomes (sex chromosomes at the top and major autosomes below) with the 4th dot chromosome omitted for brevity. We first crossed a male carrying the mutation (depicted with a horizontal line and a star) balanced over a balancer chromosome (black) to females from Dahomey (its genome colored in blue). From this cross we took mutant sons that we mated to Dahomey females. From the next cross onwards we took mutant daughters which we crossed to Dahomey males. This procedure was repeated in parallel for all 20 mutations, and replaced the mutations’ original genetic background (orange) with that of Dahomey. (B) Cross to produce focal mutant and wildtype females used in the experiment (observe that mutant and wildtype females were produce by the same parents). (C) Relative age-specific fecundity of each mutation was tested by hosting 33 mutant and 33 wildtype females with 33 males (marked with ebony [e]) in a single vial. Flies were transferred to a fresh vial every 1–2 days. At day 5 of adulthood 25 mutant and 25 wildtype females were randomly sorted out under light CO2 anesthesia and placed in separate flasks with a lid filled with food on the inside. Excess females and males were stored in separate vials. The 25 mutant and wildtype females laid eggs in the bottles over a 24 h period (food was replaced once). After egg-laying all flies were placed into a common vial and again transferred to fresh vials every 1–2 days. This procedure was repeated over five weeks, with fecundity measures taken 3 times 2 weeks apart. Males were replaced at a regular interval with respect to the fecundity assays, so that females had experienced males of the same age before eac [file 12915_2020_858_MOESM1_ESM.pptx]

## Slide 1
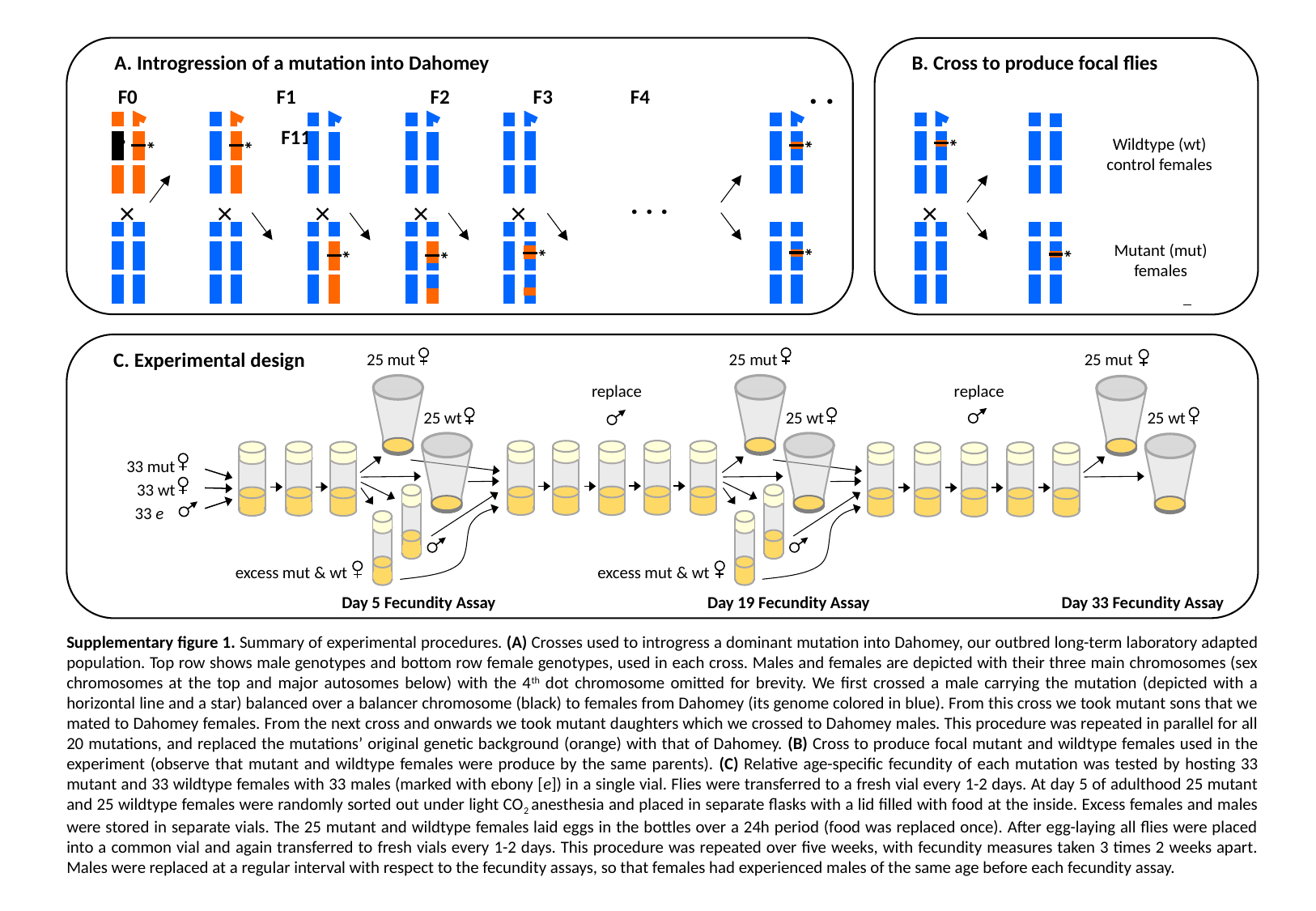

B. Cross to produce focal flies
A. Introgression of a mutation into Dahomey
F0	 F1	 F2	 F3 	 F4	 . . .	 F11
*
*
*
*
. . .
×
×
×
×
×
×
*
*
*
*
*
Wildtype (wt) control females
Mutant (mut) females
C. Experimental design
25 mut
25 wt
excess mut & wt
25 mut
25 mut
25 wt
excess mut & wt
replace
replace
25 wt
33 mut
33 wt
33 e
Day 19 Fecundity Assay
Day 33 Fecundity Assay
Day 5 Fecundity Assay
Supplementary figure 1. Summary of experimental procedures. (A) Crosses used to introgress a dominant mutation into Dahomey, our outbred long-term laboratory adapted population. Top row shows male genotypes and bottom row female genotypes, used in each cross. Males and females are depicted with their three main chromosomes (sex chromosomes at the top and major autosomes below) with the 4th dot chromosome omitted for brevity. We first crossed a male carrying the mutation (depicted with a horizontal line and a star) balanced over a balancer chromosome (black) to females from Dahomey (its genome colored in blue). From this cross we took mutant sons that we mated to Dahomey females. From the next cross and onwards we took mutant daughters which we crossed to Dahomey males. This procedure was repeated in parallel for all 20 mutations, and replaced the mutations’ original genetic background (orange) with that of Dahomey. (B) Cross to produce focal mutant and wildtype females used in the experiment (observe that mutant and wildtype females were produce by the same parents). (C) Relative age-specific fecundity of each mutation was tested by hosting 33 mutant and 33 wildtype females with 33 males (marked with ebony [e]) in a single vial. Flies were transferred to a fresh vial every 1-2 days. At day 5 of adulthood 25 mutant and 25 wildtype females were randomly sorted out under light CO2 anesthesia and placed in separate flasks with a lid filled with food at the inside. Excess females and males were stored in separate vials. The 25 mutant and wildtype females laid eggs in the bottles over a 24h period (food was replaced once). After egg-laying all flies were placed into a common vial and again transferred to fresh vials every 1-2 days. This procedure was repeated over five weeks, with fecundity measures taken 3 times 2 weeks apart. Males were replaced at a regular interval with respect to the fecundity assays, so that females had experienced males of the same age before each fecundity assay.
