# Supplementary material for: Deleterious mutations show increasing negative effects with age in Drosophila melanogaster
Source: BMC Biol. 2020 Sep 30;18:128. doi: 10.1186/s12915-020-00858-5 (PMC7526172; doi:10.1186/s12915-020-00858-5)
Supplement: Supplementary file 2 — Additional file 2: Figure S2. Correlation between relative fecundity at day 5 (ln [mut/wt]) and relative survival at day 33 (ln[mut/wt]). Circles indicate deleterious mutations with an increasing negative effect on fecundity with age, triangles indicate deleterious mutations without an increasing negative effect on fecundity with age, and squares indicate mutations for which no deleterious effect on fecundity could be detected. Associations are tested with Kendall’s tau (τ > 0.51 and p < 0.005, irrespective of whether we use the 14 aging inducing, the 16 deleterious, or all 20 mutations). Table S1. Difference in relative fecundity between wildtype and mutant (s = 1 - mut/wt) for each mutation, either only taking early-life fecundity into account, or summing over all three ages fecundity measures were taken from. Estimates are presented together with their 95% credibility interval and associated p-value. The estimation was done in a GLMM with a Poisson error distribution and log link function; positive values indicate a deleterious effect of the mutation. Table S2. Estimated coefficient for the rate of aging on fecundity for each mutation (Coeff), presented together with 95% credibility interval and associated p-value. The estimation was done in a GLMM with a binomial error distribution and logit link function where age was treated as a covariate. Negative values indicate faster aging for the mutation compared to wildtype. Table S3. Aging, estimated as the relative difference (Diff – negative estimates indicate faster mutant aging) in fecundity between mutant and wildtype between two time points, along with 95% credibility interval and associated p-value. The estimation was done in a GLMM with a binomial error distribution and logit link function where age was treated as a factor. Table S4. Summary of the number of females [minimum, median, maximum] assayed for fecundity per vial for each time point. Note that we always assayed the same number of mutant and wildtype [file 12915_2020_858_MOESM2_ESM.docx]

**Additional file 2**

**Figure S2:** Correlation between relative fecundity at day 5 and relative survival at day 33.

**Table S1:** Difference in relative fecundity between wildtype and mutant.

**Table S2:** Estimated coefficient for the rate of ageing on fecundity for each mutation.

**Table S3:** Ageing of mutant relative to wildtype across two ages.

**Table S4.** Summary of the number of females assayed for fecundity per vial for each time point.

**
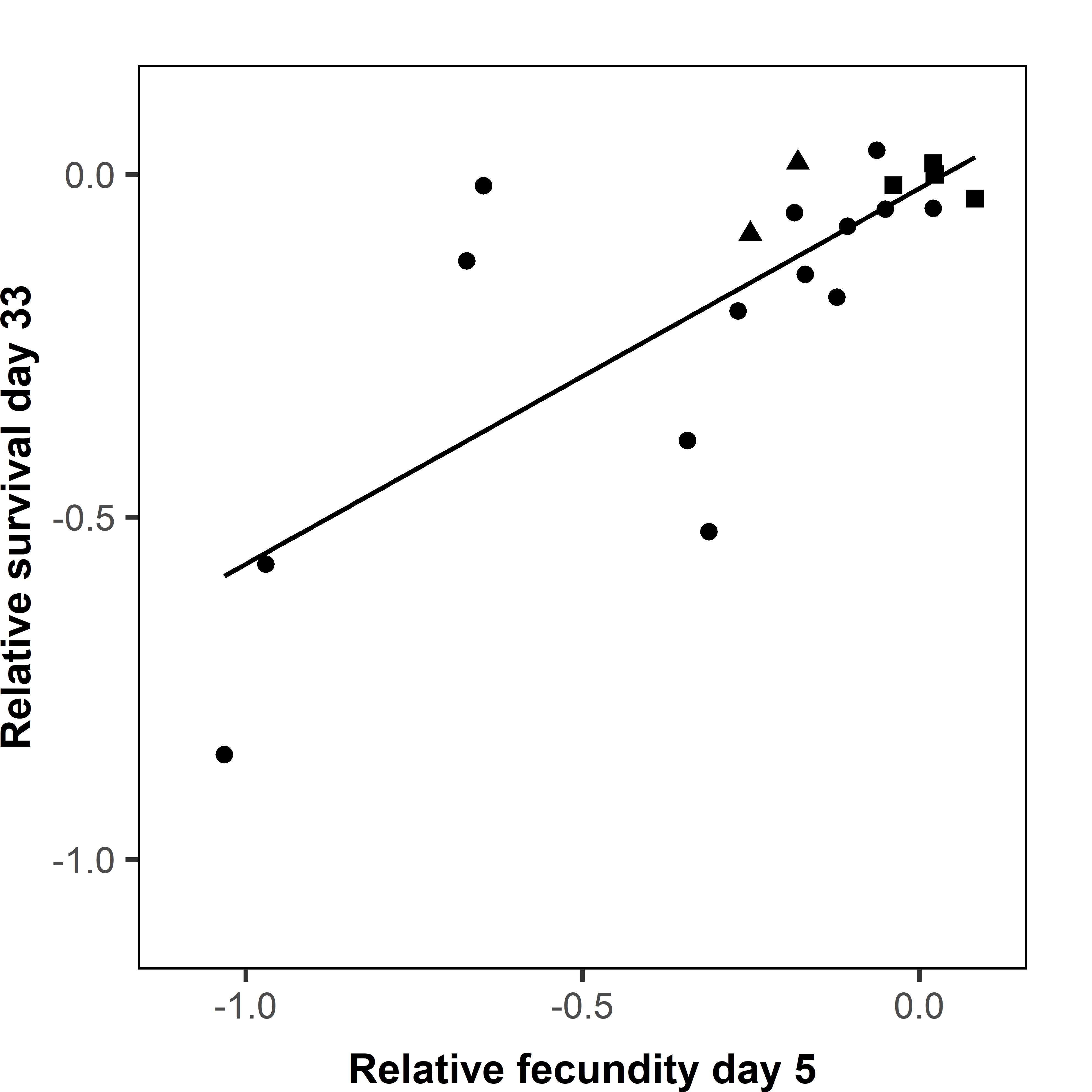
**

**Figure S2.** Correlation between relative fecundity at day 5 (ln[mut/wt]) and relative survival at day 33 (ln[mut/wt]). Circles indicate deleterious mutations with an increasing negative effect on fecundity with age, triangles indicate deleterious mutations without an increasing negative effect on fecundity with age, and squares indicate mutations for which no deleterious effect on fecundity could be detected. Associations are tested with Kendall’s tau (τ > 0.51 and p < 0.005, irrespectively of if we use the 14 ageing inducing, the 16 deleterious, or all 20 mutations).

**Table S1.** Difference in relative fecundity between wildtype and mutant (*s* = 1 - mut/wt) for each mutation, either only taking early-life fecundity into account, or summing over all three ages fecundity measures were taken from. Estimates are presented together with their 95% credibility interval and associated p-value. The estimation was done in a GLMM with a Poisson error distribution and log link function, positive values indicate a deleterious effect of the mutation.

|  | Early-life fecundity (day 5) | | | |  | Fecundity (days 5, 19 & 33) | | | |
| --- | --- | --- | --- | --- | --- | --- | --- | --- | --- |
| Mutation | *s* | 2.5% | 97.5% | *p_mcmc_* |  | *s* | 2.5% | 97.5% | *p_mcmc_* |
| *Bl* | 0.022 | -0.004 | 0.048 | 0.106 |  | -0.013 | -0.038 | 0.011 | 0.307 |
| *Bsb* | 0.142 | 0.107 | 0.176 | <**0.001** |  | 0.097 | 0.068 | 0.124 | <**0.001** |
| *BwD* | 0.021 | -0.012 | 0.052 | 0.208 |  | 0.021 | -0.008 | 0.050 | 0.147 |
| *Dfd* | 0.478 | 0.452 | 0.503 | <**0.001** |  | 0.541 | 0.523 | 0.558 | <**0.001** |
| *Dr* | 0.163 | 0.140 | 0.186 | <**0.001** |  | 0.191 | 0.171 | 0.211 | <**0.001** |
| *Frd* | 0.601 | 0.587 | 0.616 | <**0.001** |  | 0.634 | 0.623 | 0.646 | <**0.001** |
| *Gl* | 0.276 | 0.253 | 0.298 | <**0.001** |  | 0.386 | 0.369 | 0.403 | <**0.001** |
| *H2* | 0.091 | 0.067 | 0.114 | <**0.001** |  | 0.124 | 0.104 | 0.144 | <**0.001** |
| *If* | 0.201 | 0.173 | 0.228 | <**0.001** |  | 0.201 | 0.176 | 0.224 | <**0.001** |
| *Ki* | 0.079 | 0.050 | 0.107 | <**0.001** |  | 0.160 | 0.138 | 0.182 | <**0.001** |
| *L* | 0.181 | 0.144 | 0.217 | <**0.001** |  | 0.242 | 0.214 | 0.268 | <**0.001** |
| *Ly* | 0.602 | 0.589 | 0.615 | <**0.001** |  | 0.614 | 0.602 | 0.626 | <**0.001** |
| *nw* | 0.275 | 0.251 | 0.299 | <**0.001** |  | 0.314 | 0.293 | 0.335 | <**0.001** |
| *Pin* | 0.222 | 0.189 | 0.253 | <**0.001** |  | 0.250 | 0.226 | 0.274 | <**0.001** |
| *Pri* | 0.003 | -0.026 | 0.031 | 0.823 |  | 0.038 | 0.014 | 0.062 | **0.002** |
| *Pu* | 0.007 | -0.017 | 0.030 | 0.551 |  | -0.002 | -0.024 | 0.019 | 0.824 |
| *Rap* | 0.511 | 0.486 | 0.535 | <**0.001** |  | 0.490 | 0.470 | 0.509 | <**0.001** |
| *Sb* | 0.065 | 0.038 | 0.092 | <**0.001** |  | 0.082 | 0.059 | 0.105 | <**0.001** |
| *Sna* | -0.104 | -0.139 | -0.070 | <**0.001** |  | -0.093 | -0.124 | -0.063 | <**0.001** |
| *Wg* | 0.075 | 0.042 | 0.106 | <**0.001** |  | 0.088 | 0.060 | 0.116 | <**0.001** |

**Table S2.** Estimated coefficient for the rate of ageing on fecundity for each mutation (*Coeff*), presented together with 95% credibility interval and associated p-value. The estimation was done in a GLMM with a binomial error distribution and logit link function where age was treated as a covariate. Negative values indicate faster ageing for the mutation compared to wildtype.

| Mutation | *Coeff* | 2.5% | 97.5% | *p_mcmc_* |
| --- | --- | --- | --- | --- |
| *Bsb[1]* | 0.063 | 0.018 | 0.108 | **0.006** |
| *Dfd[1]* | -0.280 | -0.339 | -0.221 | <**0.001** |
| *Dr[1]* | -0.140 | -0.181 | -0.098 | <**0.001** |
| *Frd[1]* | -0.419 | -0.487 | -0.354 | <**0.001** |
| *Gl[1]* | -0.620 | -0.678 | -0.563 | <**0.001** |
| *H[2]* | -0.269 | -0.327 | -0.210 | <**0.001** |
| *Kr[If-1]* | -0.014 | -0.068 | 0.040 | 0.620 |
| *Ki[1]* | -0.286 | -0.329 | -0.242 | <**0.001** |
| *L[rm]* | -0.205 | -0.261 | -0.150 | <**0.001** |
| *Ly[1]* | -0.226 | -0.304 | -0.150 | <**0.001** |
| *nw[B]* | -0.282 | -0.348 | -0.217 | <**0.001** |
| *Pin[1]* | -0.128 | -0.177 | -0.079 | <**0.001** |
| *Pri[1]* | -0.124 | -0.167 | -0.082 | <**0.001** |
| *Rap1[1]* | -0.060 | -0.114 | -0.006 | **0.029** |
| *Sb[1]* | -0.099 | -0.152 | -0.045 | <**0.001** |
| *Wg[Sp-1]* | -0.063 | -0.116 | -0.010 | **0.020** |

**Table S3.** Ageing, estimated as the relative difference (*Diff* – negative estimates indicate faster mutant ageing) in fecundity between mutant and wildtype between two time points, along with 95% credibility interval and associated p-value. The estimation was done in a GLMM with a binomial error distribution and logit link function where age was treated as a factor.

|  | Ageing day 5 to 33 | | | |  | Ageing day 5 to 19 | | | |  | Ageing day 19 to 33 | | | |
| --- | --- | --- | --- | --- | --- | --- | --- | --- | --- | --- | --- | --- | --- | --- |
| Mutation | *Diff* | 2.5% | 97.5% | *p_mcmc_* |  | *Diff* | 2.5% | 97.5% | *p_mcm_*_c_ |  | *Diff* | 2.5% | 97.5% | *p_mcmc_* |
| *Bsb[1]* | 0.061 | -0.036 | 0.161 | 0.218 |  | 0.143 | 0.072 | 0.214 | <**0.001** |  | -0.082 | -0.191 | 0.025 | 0.129 |
| *Dfd[1]* | -0.621 | -0.762 | -0.486 | <**0.001** |  | -0.221 | -0.310 | -0.132 | <**0.001** |  | -0.400 | -0.550 | -0.250 | <**0.001** |
| *Dr[1]* | -0.359 | -0.465 | -0.254 | <**0.001** |  | -0.076 | -0.141 | -0.012 | **0.020** |  | -0.283 | -0.403 | -0.166 | <**0.001** |
| *Frd[1]* | -2.202 | -2.578 | -1.854 | <**0.001** |  | -0.192 | -0.269 | -0.116 | <**0.001** |  | -2.010 | -2.390 | -1.657 | <**0.001** |
| *Gl[1]* | -1.467 | -1.652 | -1.292 | <**0.001** |  | -0.551 | -0.622 | -0.481 | <**0.001** |  | -0.916 | -1.106 | -0.730 | <**0.001** |
| *H[2]* | -0.729 | -0.961 | -0.504 | <**0.001** |  | -0.237 | -0.306 | -0.170 | <**0.001** |  | -0.492 | -0.731 | -0.260 | <**0.001** |
| *Kr[If-1]* | -0.094 | -0.229 | 0.040 | 0.171 |  | 0.046 | -0.038 | 0.130 | 0.283 |  | -0.140 | -0.290 | 0.011 | 0.068 |
| *Ki[1]* | -0.664 | -0.768 | -0.564 | <**0.001** |  | -0.187 | -0.254 | -0.118 | <**0.001** |  | -0.478 | -0.591 | -0.363 | <**0.001** |
| *L[rm]* | -0.498 | -0.631 | -0.367 | <**0.001** |  | -0.129 | -0.211 | -0.048 | **0.002** |  | -0.369 | -0.510 | -0.228 | <**0.001** |
| *Ly[1]* | -0.872 | -1.199 | -0.562 | <**0.001** |  | -0.151 | -0.244 | -0.059 | **0.001** |  | -0.721 | -1.058 | -0.397 | <**0.001** |
| *nw[B]* | -0.686 | -0.856 | -0.517 | <**0.001** |  | -0.211 | -0.300 | -0.121 | <**0.001** |  | -0.475 | -0.660 | -0.289 | <**0.001** |
| *Pin[1]* | -0.378 | -0.492 | -0.264 | <**0.001** |  | -0.014 | -0.088 | 0.060 | 0.713 |  | -0.365 | -0.487 | -0.242 | <**0.001** |
| *Pri[1]* | -0.264 | -0.368 | -0.161 | <**0.001** |  | -0.113 | -0.176 | -0.050 | **0.001** |  | -0.151 | -0.268 | -0.034 | **0.009** |
| *Rap1[1]* | -0.467 | -0.602 | -0.333 | <**0.001** |  | 0.292 | 0.208 | 0.375 | <**0.001** |  | -0.759 | -0.902 | -0.616 | <**0.001** |
| *Sb[1]* | -0.383 | -0.569 | -0.197 | <**0.001** |  | -0.060 | -0.121 | 0.001 | 0.054 |  | -0.323 | -0.515 | -0.133 | **0.001** |
| *Wg[Sp-1]* | -0.143 | -0.272 | -0.016 | **0.029** |  | -0.048 | -0.136 | 0.040 | 0.276 |  | -0.095 | -0.246 | 0.051 | 0.208 |

**Table S4.** Summary of the number of females [minimum, median, maximum] assayed for fecundity per vial for each time point. Note that we always assayed the same number of mutant and wildtype females from a vial at each time point. *For *Kr[If-1]* and *H[2]* one vial each a was by mistake started with fewer than 33 mutant and wildtype females.

| Mutation | Day 5 | Day 19 | Day 33 |
| --- | --- | --- | --- |
| *Bl[1]* | [25,25,25] | [25,25,25] | [23,25,25] |
| *Bsb[1]* | [25,25,25] | [25,25,25] | [24,25,25] |
| *bw[D]* | [25,25,25] | [25,25,25] | [23,25,25] |
| *Dfd[1]* | [25,25,25] | [25,25,25] | [19,25,25] |
| *Dr[1]* | [25,25,25] | [25,25,25] | [18,25,25] |
| *Frd[1]* | [25,25,25] | [21,25,25] | [7,12,18] |
| *Gl[1]* | [25,25,25] | [25,25,25] | [13,21,25] |
| *H[2]** | [22,25,25] | [18,25,25] | [16,25,25] |
| *Ki[If-1]** | [17,25,25] | [16,25,25] | [15,25,25] |
| *Ki[1]* | [25,25,25] | [25,25,25] | [17,25,25] |
| *L[rm]* | [25,25,25] | [25,25,25] | [18,25,25] |
| *Ly[1]* | [25,25,25] | [23,25,25] | [7,15,22] |
| *Nw[B]* | [25,25,25] | [25,25,25] | [12,19,25] |
| *Pin[1]* | [25,25,25] | [22,25,25] | [19,24,25] |
| *Pri[1]* | [25,25,25] | [25,25,25] | [25,25,25] |
| *Pu[2]* | [25,25,25] | [25,25,25] | [25,25,25] |
| *Rap[1]* | [25,25,25] | [25,25,25] | [22,25,25] |
| *Sb[1]* | [25,25,25] | [25,25,25] | [25,25,25] |
| *Sna[Sco]* | [25,25,25] | [25,25,25] | [20,25,25] |
| *Wg[Sp-1]* | [25,25,25] | [25,25,25] | [22,25,25] |
